# Soil Native C/N Ratio Affects Diazotrophic Bacterial Composition and N Fixation by Regulating SOC Distribution in Soil Particles After Residue Incorporation

**DOI:** 10.3390/microorganisms13051104

**Published:** 2025-05-11

**Authors:** Pengfei Duan, Di Zhao, Shuqiong Yang, Jibao Chen, Zhaojin Chen, Lingling Cao

**Affiliations:** Collaborative Innovation Center of Water Security for Water Source Region of Mid-Route Project of South-North Water Diversion of Henan Province, Nanyang Normal University, Nanyang 473061, China; lf4931@126.com (D.Z.); sqyang@nynu.edu.cn (S.Y.); chenjibao2012@163.com (J.C.); zhaojin_chen@163.com (Z.C.); cao_ling2019@163.com (L.C.)

**Keywords:** soil particle, C/N, organic carbon decomposition gene, diazotrophic bacteria community, biological N fixation

## Abstract

Residue incorporation is a universally accepted agronomic practice around the world. However, the relationship between C and N turnover in soil is still not fully understood. We performed a soil microcosm incubation experiment to study the effects of residue incorporation with different C-to-N ratios (C/N) (low, 31.8; high, 84.5) on the SOC distribution, diazotrophic bacterial composition, and N accumulation in different particle size fractions (PSFs) of three types of soil parent material with different native C/N ratios. Our results demonstrate that SOC is 1.34 to 2.50 times more easily retained and accumulated in clay compared to baseline levels at the early stage of soil development. The native C/N ratio markedly affects the microbial metabolic characteristics and the SOC accumulation processes. Specifically, low C/N residue enhanced SOC accumulation in high-native-C/N soil, while the high-C/N residue maximized SOC in low-native-C/N soil. These shifts regulated the composition of the diazotrophic bacterial community, increasing the nitrogenase coding gene (*nifH*) abundance 5.57- and 8.1-fold in low-C/N residue treatments and 6.6-fold in the high-C/N residue treatment, thereby promoting soil N accumulation. Moreover, we highlight the potential role of *Bradyrhizobiaceae*, *Rhodospirillaceae*, *Micrococcaceae*, *Rhizobiaceae*, *Comamonadaceae*, *Nitrospiraceae*, and *Burkholderiaceae* in biological N fixation during pedogenesis. This study explored the coupling relationship between soil native and residue C/N and soil biological N fixation at the soil particle level, and the results suggest that residue incorporation to improve soil fertility needs to be further explored according to soil native C/N.

## 1. Introduction

Generally, high levels of nitrogen (N), phosphorus (P), potassium (K), and soil organic carbon (SOC) are critical in sustainable soil fertility and crop productivity [1]. Unlike P and K, soil C and N can accumulate during pedogenesis by photosynthesis, N fixation, and deposition in the absence of artificial influences. In agroecosystems, C sequestration is mainly achieved by plant photosynthesis and returns to the soil in the form of root exudate and residue [2], whereas N fixation is mainly achieved by diazotrophic microbial processes if there is no fertilizer input [3]. Recent meta-analyses have further highlighted that SOC responses to residue incorporation are influenced by the soil texture (e.g., silt content), total N, and land-use history [4].

Crop residues are natural soil amendments containing valuable nutrients, especially organic C, and hence they are often incorporated into soils to improve soil fertility in sustainable agriculture. Residue incorporation is recommended to farmers for improving soil structure and preventing SOC decline [5]. On a global scale, a large amount of C is sequestered annually in agricultural soils by residue incorporation [6]. Generally, N dynamics, including N fixation, are usually coupled with the decomposition of crop residues [7]. The C/N ratio of crop residues has been widely demonstrated to be important in controlling residue decomposition and soil N dynamics [8,9]. Low C/N residues increase the soil microbial biomass and respiration [10]; hence, they can promote degradation processes and stimulate N mineralization [11]. However, residues with a high C/N ratio may improve soil biological N fixation but trigger N immobilization by soil and fertilizers [12,13]. Moreover, Powlson et al. [14] found that the responses of SOC and TN to residue incorporation vary widely and are site-specific. Therefore, the SOC and soil biological N fixation under crop residue incorporation may largely depend on soil properties and residue qualities.

The C/N ratio of a crop residue markedly affects the soil properties in the initial de-composition period [15]. Furthermore, long-term different C/N crop residue incorporation can significantly change soil properties by shifting the SOC distribution and biological N fixation and consequently the N concentration [16]. Zhao et al. [17] found that the effects of crop residue on soil properties and nutrient cycling subsided almost completely within 6 months and pointed out that the effects of crop residue incorporation varied with the planting season. However, the temporal, especially long-term, effects of crop residue incorporation on microbial process-related soil C turnover and biological N fixation have been less investigated. Therefore, belowground biological processes, especially their effects on soil nutrient pools with residue incorporation, still need further exploration.

To explore how soil C turnover and biological N fixation respond to residue incorporation, we established a three-year soil microcosm incubation experiment using three types of red soil for parent material (high and low native C/N) and two types of residue (high and low C/N). We selected the soil parent materials due to the limited available nutrients and slow nutrient accumulation processes [18]. The objectives of this study were (i) to compare the distribution of soil C in different PSFs; (ii) to compare the variations in soil N accumulation and the diazotrophic bacterial community after residue incorporation. We hypothesized that native soil properties would have different impacts on residue decomposition and SOC distribution in soil; hence, they would have different effects on soil biological N fixation and N accumulation.

## 2. Materials and Methods

### 2.1. Soil Samples

Soil parent materials comprise loose debris formed by the weathering of bare rocks. Quaternary red clay soil (CS), granite soil (GS), and purple sandy shale (PS) are three types of soil parent materials that represent the three major soil parent materials of the agricultural soils in Southern China [18]. Parent materials were collected form Hunan Province (111°06′–112°21′ E, 24°39′–26°51′ N) in May 2020. All samples were passed through a 2 mm sieve. Subsamples used for measuring physicochemical properties were air-dried, and other subsamples used for incubation were temporarily stored at room temperature until use.

### 2.2. Experimental Design, Soil Sampling, and Analysis

Soil microcosms were prepared as described by Xun et al. [19]. Nine replicate microcosms were established for each type of soil, and each microcosm was constructed by placing 250 g of soil into a 500 mL bottle. Next, sterile water was added to maintain a constant moisture level (30% of field capacity), and the samples were pre-incubated at 25 °C in the dark for 2 weeks before residue incorporation. A rice residue with a high C/N ratio (84.5:1) and a green manure crop residue with a low C/N ratio (31.8:1) were used for residue incorporation. The residue was provided in the form of residue powder at 4 g kg^−1^ (0.4%) and thoroughly mixed with soil. Three treatments were established for each type of soil in this study: high-C/N-ratio residue incorporation (H, rice residue incorporation), low-C/N-ratio residue incorporation (L, green manure crop residue incorporation), and mixed-residue incorporation (M, rice residue and manure crop residue incorporation, 50%:50%). The microcosms were incubated at 25 °C at constant moisture (45% of field capacity) for 3 years. The residues were added every 6 months during the incubation period, and the samples were stirred only at residue application to simulate agricultural management.

After a three-year incubation period, the soils were gently broken apart along the natural break points and sieved (<5 mm) to remove visible organic debris. Then, subsamples were collected for DNA extraction and the remaining samples were fractionated by ultrasound disruption in distilled water. Three different PSFs, including a 63–2000 μm size fraction (sand), a 2–63 μm size fraction (silt), and a <2 μm size fraction (clay), were collected. All PSFs were freeze-dried before soil property measurements were taken. Soil TN and SOC were measured following previously described methods [20]. The activity of β-glucosidase (EC 3.2.1.21) was measured using pnitrophenyl glycoside as the substrate [21].

### 2.3. DNA Extraction and qPCR

Total soil DNA was extracted using a PowerSoil DNA Isolation Kit (Mo Bio Laboratories Inc., Carlsbad, CA, USA). DNA quality was tested using a NanoDrop ND-2000 spectrophotometer (NanoDrop, ND2000, ThermoScientific, Wilmington, DE, USA) based on the 260/280 nm and 260/230 nm absorbance ratios. Three successive DNA extractions of each sample were pooled to minimize the DNA extraction bias. The extracted DNA samples were used for analyzing bacterial community compositions and measuring *nifH* gene abundances.

The *nifH* gene abundances were investigated using quantitative real-time PCR with an ABI 7500 real-time PCR system (Applied Biosystems, Carlsbad, CA, USA). The following primer set was used: polF, 5′-TGCGAYCCSAARGCBGACTC-3′, and polR, 5′-ATSGCCATCATYTCRCCGGA-3′ [3]. For standard curve construction, a plasmid standard containing a cloned *nifH* gene fragment (370 bp) was generated via TOPO-TA cloning (Thermo Fisher Scientific). The plasmid was then linearized and serially diluted (10^4^ to 10^8^ copies μL^−1^) to establish the standard curve. For *nifH* gene quantification, the reactions were conducted in 20 μL volumes containing 10 μL of 2× SYBR Premix Ex Taq^TM^ (TaKaRa Bio, Otsu, Shiga, Japan), 0.4 μL each of forward and reverse primers (10 μM; polF/polR), 1–2 μL of template DNA (10–50 ng), and molecular-grade water to adjust the final volume. Thermocycling conditions included an initial denaturation at 95 °C for 5 min, followed by 40 cycles of 95 °C for 15 s, 60 °C for 30 s, and 72 °C for 30 s, with a final melt curve analysis (60–95 °C, 0.3 °C/s increment) to confirm amplification specificity [22].

### 2.4. Amplicon Sequencing

The V4 hypervariable region of the bacterial 16S rRNA gene was amplified using the primers 515F (5′-GTGCCAGCMGCCGCGGTAA-3′) and 806R (5′-GGACTACHVGGGTWTCTAAT-3′) [21] to assess bacterial communities. The PCR amplicons were combined in equimolar ratios, and sequencing was conducted by Bion Biotechnology Co., Ltd. (Nanjing, China) on an Illumina MiSeq platform with separate sequencing runs for the 16S rRNA gene amplicon pools. The sequencing data were processed using the UPARSE pipeline (http://drive5.com/usearch/manual/uparse_pipeline.html, accessed on 10 March 2024) as previously described [23]. Briefly, the raw sequences were subjected to quality control. The singleton and chimeric sequences were removed after dereplication, and the remaining sequences were categorized into operational taxonomic units (OTUs) with 97% similarity and then assigned taxonomy using the Silva database (Release 128) (https://www.arb-silva.de/, accessed on 5 June 2024). The potential metabolic functions were predicted by PICRUSt2.

### 2.5. Statistical Analysis

The relative percentage of each phylum (family) was used as the relative abundance of the phylum and family for the pyrosequencing data. Duncan’s multiple-comparison analysis was performed to compare the parameters among treatments. The correlations were calculated using Spearman’s rank correlation. All statistical analyses, including Shannon’s diversity index and non-metric multidimensional scaling (NMDS) analysis, were performed using Vegan package (v.2.4-1) in R software package (version 3.3.2).

## 3. Results

### 3.1. Soil Native C/N Affects SOC Distribution Under Residue Incorporation

The three soil parent materials, though all classified as Ferralic Cambisol under the World Reference Base system, exhibited marked heterogeneity in particle size fractions (PSFs) (Table 1). Specifically, CS and PS displayed silt-dominated profiles, constituting 64.0% ± 1.2% and 74.4% ± 1.7% of total particles, respectively, whereas GS demonstrated a bimodal distribution with sand (42.2% ± 3.1%) and silt (45.1% ± 1.4%) as co-dominant fractions. This divergence likely originated from differential weathering intensities and transport mechanisms during parent material formation.

The original SOC concentrations across all particle fractions remained exceptionally low, ranging from 0.41 g kg^−1^ to 4.55 g kg^−1^, consistent with highly weathered tropical soils. Notably, for all of the three parent materials, the SOC was mainly enriched in clay (3.52~4.55 g kg^−1^), less enriched in silt (1.86~2.48 g kg^−1^), and least enriched in sand (0.41~1.65 g kg^−1^).

Total nitrogen (TN) dynamics revealed parallel yet distinct trends, with residue amendments significantly elevating TN concentrations in clay and silt fractions. In addition, residue incorporation induced significant SOC accumulation (*p* < 0.05, *t*-test) across all particle size classes, with the maximum sequestration observed in clay fractions (1.34 to 2.50 times the baseline levels). However, the spatial distribution of TN and SOC within particle fractions showed treatment-specific patterns contingent upon initial C/N ratios. Notably, in clay fractions, the PS_H treatment achieved higher TN concentrations (1.06 ± 0.09 g kg^−1^) than PS_L (0.82 ± 0.06 g kg^−1^) despite a lower residue N input, implying an enhanced nitrogen retention efficiency in low C/N systems. The hierarchical TN accumulation pattern (CS_H < CS_L, GS_H < GS_L) further corroborated the critical role of residue quality in nitrogen cycling. Mechanistically, the preferential association of TN with finer particles likely stems from organo-mineral complexation and physical protection within micro-aggregates. Furthermore, low C/N residues preferentially enhanced clay-associated SOC in CS (8.18 ± 0.11 g kg^−1^) and GS (13.07 ± 0.16 g kg^−1^), which had inherently higher parent material C/N ratios (CS_PM, 24.1 ± 2.1; GS_PM, 25.4 ± 3.2). Conversely, high-C/N residues maximized clay SOC accumulation in PS (10.47 ± 0.15 g kg^−1^), where the native parent material C/N was significantly lower (PS_PM, 7.7 ± 0.4).

### 3.2. Residue Incorporation Alters Soil Bacterial Composition

**Figure 1:**
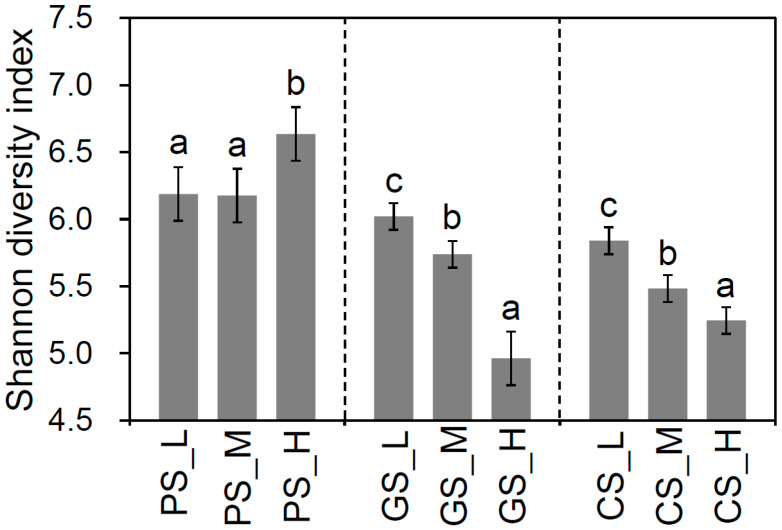
Shannon’s diversity indices for all residue incorporation treatments. CS, Quaternary red clay soil; GS, granite soil; PS, purple sandy shale; PM, parental material; L, low-C/N-ratio residue incorporation; H, high-C/N-ratio residue incorporation; M, mixed-residue incorporation with low- and high-C/N-ratio residues. Different letters indicate significant differences (*p* value < 0.05) between residue incorporation treatments within each type of parent material according to Duncan’s multiple-comparison test.

Residue incorporation induced significant variations in bacterial community diversity and compositional profiles across soil parent materials. Shannon’s diversity indices exhibited treatment-dependent patterns contingent upon parent material characteristics (Figure 1). The maximum value for Shannon’s diversity was observed in PS_H (6.63 ± 0.21), contrasting with CS and GS systems where low-C/N treatments (CS_L, 5.84 ± 0.11; GS_L, 6.02 ± 0.13) supported richer communities than high-C/N treatments (CS_H, 5.25 ± 0.13; GS_H, 4.96 ± 0.20). This inverse relationship between residue quality and diversity in CS/GS systems may reflect the competitive exclusion of oligotrophic lineages under rapid carbon mineralization regimes.

**Figure 2:**
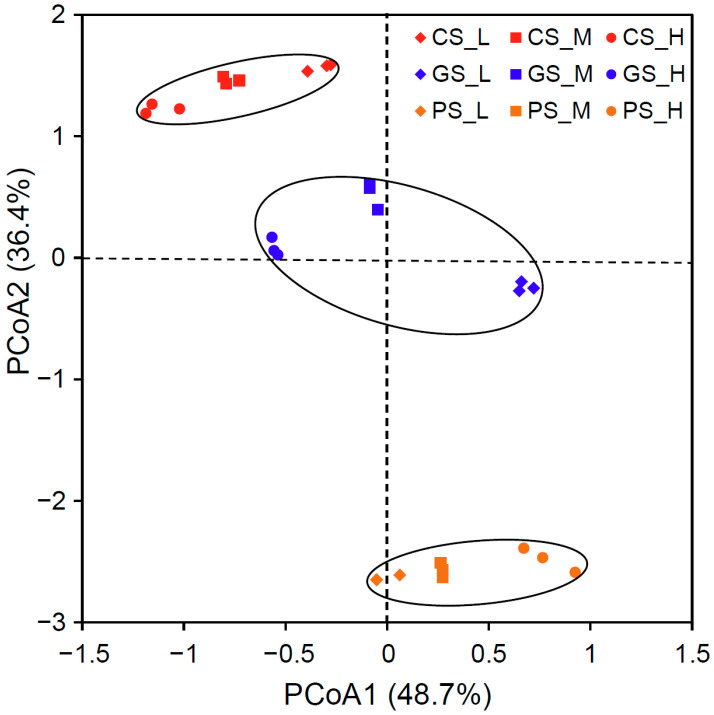
Non-metric multidimensional scaling (NMDS) analysis for bacterial community. CS, Quaternary red clay soil; GS, granite soil; PS, purple sandy shale; PM, parental material; L, low-C/N-ratio residue incorporation; H, high-C/N-ratio residue incorporation; M, mixed-residue incorporation with low- and high-C/N-ratio residues.

Principal coordinates analysis (PCoA) revealed distinct separation patterns along orthogonal axes (Figure 2), with the first principal coordinate explaining 48.7% of the total variance and segregating communities primarily by residue C/N ratio (Mantel test r = 0.592, *p* < 0.001). This axis differentiated high-C/N (H) and low-C/N (L) treatments into discrete clusters, suggesting that the stoichiometric properties of organic inputs fundamentally restructured microbial assemblages. The second principal coordinate, accounting for 36.4% of variance, partitioned communities according to parent material type (CS, GS, PS) (Mantel test r = 0.376, *p* < 0.001), indicating that inherent pedogenic differences modulated treatment-specific responses.

**Figure 3:**
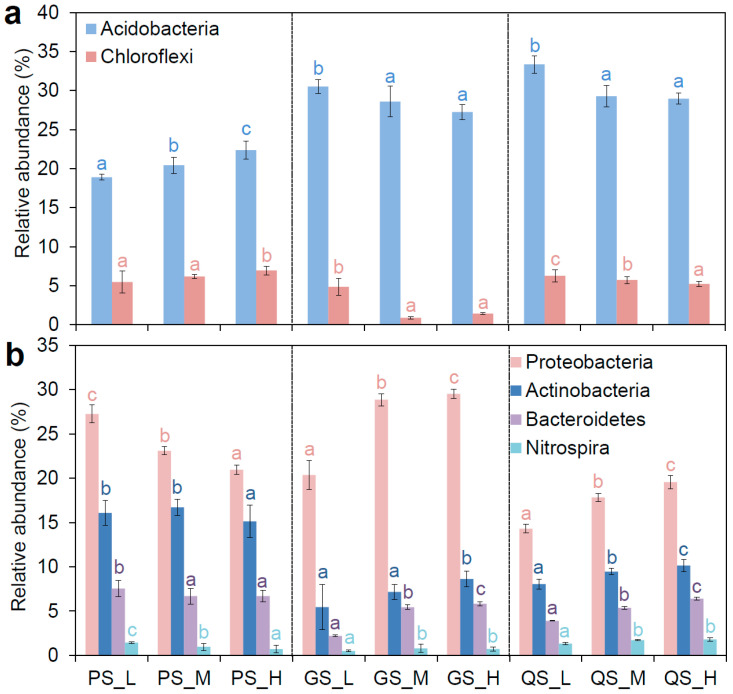
(**a**) Relative abundance of Acidobacteria and Chloroflexi in all residue incorporation treatments. (**b**) Relative abundance of Proteobacteria, Actinobacteria, Bacteroidetes, and Nitrospira in all residue incorporation treatments. CS, Quaternary red clay soil; GS, granite soil; PS, purple sandy shale; PM, parental material; L, low-C/N-ratio residue incorporation; H, high-C/N-ratio residue incorporation; M, mixed-residue incorporation with low- and high-C/N-ratio residues. Different letters indicate significant differences (*p* value < 0.05) between residue incorporation treatments within each type of parent material according to Duncan’s multiple-comparison test.

Phylum-level compositional shifts demonstrated reciprocal abundance patterns between treatments. Generally, the Acidobacteria (18.89~33.36%) and Proteobacteria (14.31~28.86%) were the dominate phyla in the communities, followed by the Actinobacteria (7.16~16.70%). Interestingly, we found that the relative abundances of Acidobacteria (22.37% ± 1.18%) and Chloroflexi (6.94% ± 0.57%) were higher in PS_H than those in PS_M and PS_L (Figure 3a). However, they were more abundant in CS_L than those in CS_M and CS_H, and more abundant in GS_L than those in GS_M and GS_H. These distributions of Acidobacteria and Chloroflexi are consistent with their oligotrophic survival strategies under high-C/N conditions. In contrast, the copiotrophic taxa including Proteobacteria (14.31~28.86%), Actinobacteria (6.61~16.70%), Bacteroidetes (2.19~7.55%) and Nitrospira (0.50~1.80%) were in greater abundances in CS_H and GS_H than the corresponding lower C/N residue incorporation treatments (Figure 3b). However, these copiotrophic taxa were enriched in PS_L (Proteobacteria, 27.25% ± 1.01%; Actinobacteria, 16.07% ± 3.42%; Bacteroidetes, 7.55% ± 0.95%; Nitrospira, 1.44% ± 0.12%). These results suggest that the substrate quality selectively regulates functional guilds among these phyla.

**Figure 4:**
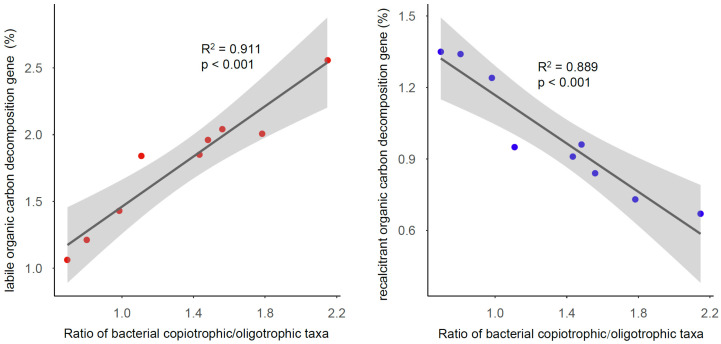
The relationships between the ratio of bacterial copiotrophic/oligotrophic taxa and the labile (**left**) or recalcitrant (**right**) organic carbon decomposition gene. The ratio of bacterial copiotrophic/oligotrophic taxa was calculated by dividing the total relative abundance of Proteobacteria, Actinobacteria, Bacteroidetes, and Nitrospira by the relative abundance of Acidobacteria and Chloroflexi.

**Figure 5:**
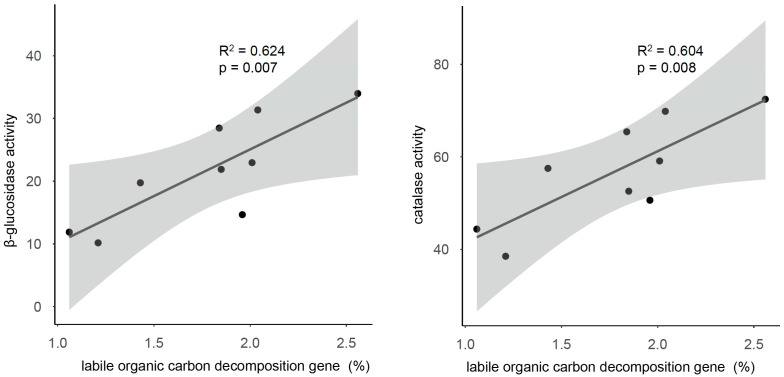
The relationships between the labile organic carbon decomposition gene and the β-glucosidase (**left**) and catalase activity (**right**).

To evaluate the effects of compositional variations on soil organic carbon decomposition processes, we collected the functional genes related to the bacterial metabolism processes by PICRUSt2, including the labile and recalcitrant organic carbon decomposition genes (Table S1). Interestingly, the relative abundance of the labile organic carbon decomposition gene was positively correlated (R^2^ = 0.911, *p* < 0.001) with the ratio of the bacterial copiotrophic/oligotrophic taxa (Figure 4), which was calculated by dividing the total relative abundance of Proteobacteria, Actinobacteria, Bacteroidetes, and Nitrospira by the relative abundance of Acidobacteria and Chloroflexi. However, the relative abundance of the recalcitrant organic carbon decomposition gene was negatively correlated (R^2^ = 0.889, *p* < 0.001) with the ratio. Importantly, the β-glucosidase and catalase activities were both positively correlated (R^2^ = 0.624, *p* = 0.007 for β-glucosidase, R^2^ = 0.604, *p* = 0.008 for catalase) with the relative abundance of the labile organic carbon decomposition gene (Figure 5).

### 3.3. Residue Incorporation Affects Diazotrophic Bacterial Composition and Soil Biological N Fixation

**Figure 6:**
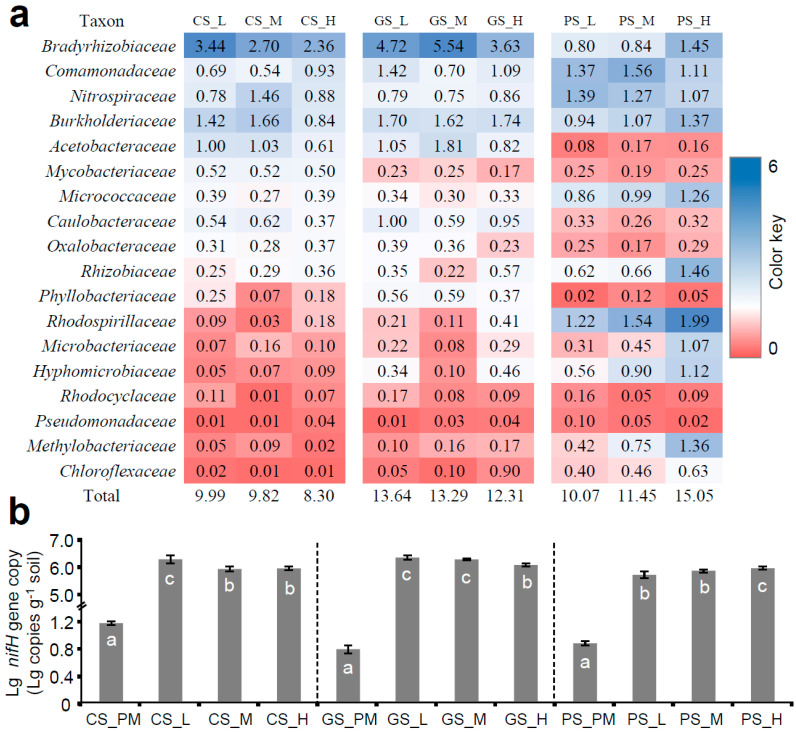
(**a**) Relative abundance (%) of N-fixing bacterial family in all residue incorporation treatments. Color scale from greatest (blue) to lowest (red) relative abundances within same type of soil. (**b**) Lg-transformed *nifH* gene copy under all residue incorporation treatments based on qPCR. Different letters indicate significant differences (*p* value < 0.05) between residue incorporation treatments within each type of parent material according to Duncan’s multiple-comparison test. CS, Quaternary red clay soil; GS, granite soil; PS, purple sandy shale; PM, parental material; L, low-C/N-ratio residue incorporation; H, high-C/N-ratio residue incorporation; M, mixed-residue incorporation with low- and high-C/N-ratio residues.

Residue incorporation shifted the composition of the soil bacterial community and hence triggered changes in the diazotrophic bacterial composition and abundance. We collected all the potential diazotrophic bacterial families and found that the most abundant diazotrophic bacterial family in CS and GS was *Bradyrhizobiaceae* (2.36~5.54%), followed by *Burkholderiaceae* (0.84~1.74%), *Comamonadaceae* (0.54~1.42%), *Acetobacteraceae* (0.61~1.81%), *Nitrospiraceae* (0.75~1.46%), etc. (Figure 6a). Moreover, the most abundant diazotrophic bacterial family in PS was *Rhodospirillaceae* (1.22~1.99%), followed by *Nitrospiraceae* (1.07~1.39%), *Burkholderiaceae* (0.94~1.37%), *Comamonadaceae* (1.11~1.56%), *Bradyrhizobiaceae* (0.80~1.45%), *Micrococcaceae* (0.86~1.26%), *Methylobacteriaceae* (0.42~1.36%), etc. These compositional disparities likely reflect differential redox conditions and carbon availability across parent materials.

Quantitative PCR analysis demonstrated treatment-specific impacts on diazotrophic abundance and *nifH* gene copy numbers (Figure 6b). Low-C/N residues significantly enhanced diazotrophic bacterial populations in CS_L (5.57 times of baseline in CS_PM) and PS_L (8.1 times of baseline in GS_PM). Conversely, GS displayed inverse trends, with high-C/N treatments eliciting a greater stimulation of the *nifH* gene copy number (6.6 times that of the baseline in PS_PM). This divergence suggests parent material physicochemical properties like the native C/N ratio and mineralogy might mediate residue quality effects on the nitrogen fixation potential.

Spearman’s correlation analysis elucidated strong associations between clay-associated nutrients and diazotrophic activity (Table 2). The TN content in clay fractions exhibited significant positive correlations with *nifH* gene abundance (R = 0.762, *p* < 0.01) and diazotrophic bacterial abundance (R = 0.885, *p* < 0.01), highlighting clay’s role as a microbial niche. Similarly, soil organic carbon (SOC) in clay showed robust correlations with *nifH* (R = 0.819, *p* < 0.01) and diazotrophs (R = 0.903, *p* < 0.01), underscoring carbon availability as a key driver of biological nitrogen fixation. These findings collectively demonstrate that residue-driven modifications to bacterial communities exert cascading effects on organic carbon metabolism and nitrogen fixation processes, with outcomes contingent upon both residue stoichiometry and parent material textural characteristics.

## 4. Discussion

It has been extensively demonstrated that residue incorporation has positive effects on SOC and TN across large variations in climate and land-use types [24,25,26]. Despite the differing soil PSFs among the three parent materials, SOC accumulation consistently favored clay over sand particles in all treatments. Previous studies have suggested that soil C and N turnover differ according to aggregate size [27,28] and soil type due to PSF compositions [29]. The accumulation of SOC in the largest aggregate sizes through the inorganic binding of silt and clay particles in pastures and forests [30] and the patterns of greater transformation of plant inputs are consistent with decreasing fraction size [31]. Additionally, Peng et al. [32] demonstrated that new SOC is preferentially incorporated into macro-aggregates but persists for a shorter time than in micro-aggregates and silt- and clay-sized aggregates using the 13C labeling technique in rare-earth oxides. Therefore, we infer that SOC is more easily retained and accumulated in clay particles during early soil development, which comprises the incipient succession of soil before forming macro-aggregates, and the continuous accumulation of SOC will consequently promote macro-aggregation and improve soil fertility [33].

We observed distinct responses in SOC decomposition and distribution across the three parent materials under high- and low-C/N residue incorporations. The SOC from the low-C/N residue was more easily accumulated in clay with a higher native C/N ratio. Simultaneously, the SOC from high-C/N residue was more easily accumulated in clay with a lower native C/N ratio. However, this accumulation is not present in sand particles. Consequently, the native C/N in clay and silt particles may reflect the decomposition of newly invested residue and the preferred appropriate C/N ratio. Therefore, the clay fraction emerged as the principal sink for both SOC and TN, accounting for up to 50.0% of the total sequestered carbon and 65.6% of the retained N. These findings underscore the necessity of substrate–soil matrix compatibility assessments when designing residue management strategies for different types of parent material. This inverse relationship suggests that the C/N stoichiometry probably governs substrate utilization, which involves microbial processes modulating C and N cycling pathways.

Decomposition and preference behaviors were mainly determined by soil microbes. Moreover, as processes such as the degradation or decomposition of SOC may release organic acids [34,35], they could further facilitate the release of nutrients such as P, K, and sulfur (S) from weathered parent materials [36]. Such processes are enhanced in micro-aggregates due to their higher surface area and ion exchange capacity compared to macro-aggregates [37,38]. Meanwhile, soil microbes tend to colonize in eutrophic environments with high SOC and mineral element concentrations. Therefore, microbiological actions, SOC accumulation and mineralization, and the release of mineral elements present concurrently and may preferentially take place in clay particles at the early stage of soil development.

Generally, soil microbial community composition is strongly influenced by SOC pool distribution, reflecting microbial substrate preferences [39]. Previous studies have discovered an oligotrophic–copiotrophic switch in the soil microbial community when nutrient availability increased [40]. In this study, we found that Shannon’s diversity for bacteria, as well as the relative abundances of copiotrophic bacteria, e.g., Nitrospira and Proteobacteria [41], was positively associated with the SOC concentration in clay [42]. Therefore, the C/N ratio was suggested to be the critical factor influencing the residue decomposition and bacterial community under residue incorporation.

The divergent responses of the oligotrophic taxa (e.g., Acidobacteria, Chloroflexi) and the copiotrophic taxa (e.g., Proteobacteria, Actinobacteria, Bacteroidetes, Nitrospira) to residue inputs align with the ecological theory predicting C/N-mediated niche partitioning [43]. A recent study corroborated that Proteobacteria abundance increased by 30–70% under low-C/N conditions (C/N = 3) compared to higher ratios (C/N = 5), with significant implications for denitrification processes [44]. However, the C/N ratios in the above study are lower than the optimal ratio of 25 for microbial metabolism. Here, C/N ratio regulation, including the lower parent material C/N ratio in PS treated with the higher C/N ratio residue, as well as the higher parent material C/N ratio in CS and GS treated with the lower C/N ratio residue, likely favors oligotrophic taxa. This trophic stratification is substantially modulated by soil parent material characteristics [18]. The higher native C/N ratios of CS and GS amplify the stimulatory effects of high-C/N residues on copiotrophs through enhanced substrate–soil stoichiometric compatibility, while the lower native C/N of PS soil favors oligotrophs under high-C/N residue inputs. That substrate–soil matrix interactions critically regulate microbial metabolic strategies has been demonstrated through the isotope tracing of carbon flow pathways [45]. These findings have significant implications for sustainable soil management. The strategic alternation of high- and low-C/N residues could optimize the trade-off between short-term nutrient mineralization and long-term carbon sequestration [14].

The C/N ratios in clay and silt particles suggested differences in the degree of SOC turnover and enrichment of microbial-processed SOC in mineral-associated fractions [46]. Here, we observed approximate C/N ratios in clay particles at the end of the incubation period, although the residue-N input was differed and limited, indicating that N accumulated with the enrichment of SOC in clay. Therefore, we speculate that the diazotrophic bacteria may contribute a significant amount of fixed N. Therefore, the abundance of the *nifH* gene and the composition of the diazotrophic bacterial community were analyzed. We found that the abundances of the *nifH* gene and diazotrophic bacteria were positively correlated with the SOC concentration in clay. However, the compositions of the diazotrophic bacterial community and the predominant diazotrophic bacteria were different in three types of parent materials due to the distinct physical structures and chemical properties, among which the soil pH and SOC concentration were the most important environmental drivers [47]. For instance, *Bradyrhizobiaceae* was observed as the most abundant diazotrophic bacteria in acidic soils [48] and could contribute more than 25% to the taxonomic composition of *nifH* gene families [49]. In contrast, *Rhodospirillaceae*, *Micrococcaceae*, and *Rhizobiaceae* increased in abundance with increasing pH [48]. Additionally, other diazotrophic families, e.g., *Comamonadaceae*, *Nitrospiraceae*, and *Burkholderiaceae*, have similar abundances in all soils. It has been demonstrated that these families belong to phyla whose functionalities are associated with carbon cycling [50]. Moreover, most of the *nifH* genes belonging to copiotrophic bacteria, such as *Burkholderiaceae*, dominate in organically cultivated soils [3]. Similarly, many members from *Comamonadaceae* are important decomposers in soils and are probably involved in P acquisition, serving as P sinks [51]. These results demonstrate that the accumulation of SOC in clay may shift the diazotrophic bacterial community and stimulate biological N fixation in soil, and, prior to that, the native C/N ratio in clay is one important determinant of SOC accumulation.

Although the connection between native and residue C/N ratios and soil C sequestration and biological N fixation has been established via a three-year period of incubation, the gaps between laboratory insights and field applications still greatly need to be addressed. Long-term field trials are essential in validating microcosm-based findings, particularly the role of clay fractions as persistent SOC and N sinks. In addition, the mechanistic links between residue stoichiometry, microbial functional traits, and nutrient fluxes require further interrogation using multi-omics approaches like metagenomics, unlocking how N fixation taxa modulate nitrogenase activity (*nifH* expression) in response to SOC–clay interactions. Moreover, the interplay between residue management and other agronomic practices like tillage could be quantified to design synergistic soil fertility strategies, enabling precision management tailored to soil parent materials and regional pedoclimatic conditions.

## 5. Conclusions

**Figure 7:**
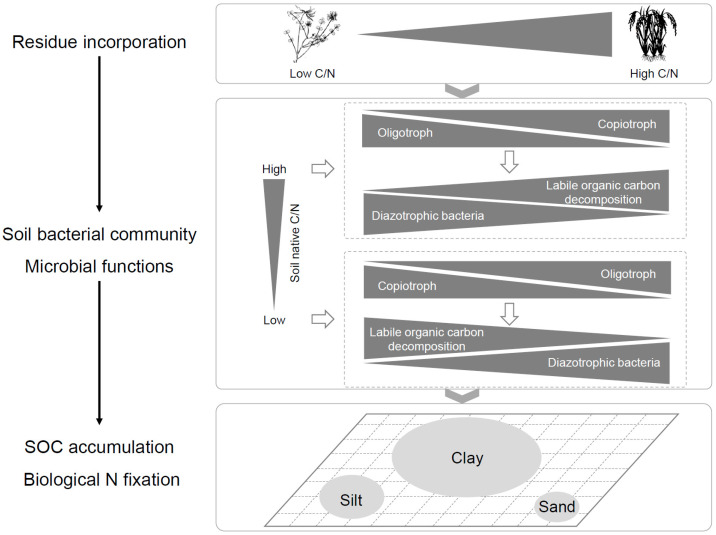
The schematic diagram illustrating the hypothesized pathways of the microbial community-triggered SOC accumulation and biological N fixation. The soil native C/N ratio and residue C/N ratio markedly affect the SOC accumulation, the composition of the diazotrophic bacterial community, and the N fixation capacity in clay.

In summary, the results for how SOC distribution and diazotrophic bacterial communities respond to residue incorporation in three types of soil parent material indicate that SOC is more easily retained and accumulated in clay in the early stage of soil development. In addition, the soil native C/N ratio markedly affects the SOC accumulation, the composition of the diazotrophic bacterial community, and the N fixation potential, which are mechanistically linked to stoichiometry-driven microbial niche partitioning (Figure 7). Our findings highlight the contribution of *Bradyrhizobiaceae*, *Rhodospirillaceae*, *Micrococcaceae*, *Rhizobiaceae*, *Comamonadaceae*, *Nitrospiraceae*, and *Burkholderiaceae* to N accumulation during soil maturation, particularly through their association with clay-associated SOC and TN pools. These results establish a critical connection between native and residue C/N ratios and soil C sequestration and biological N fixation at the particle level. Further work should explore the long-term interplay between soil-inherent and externally added C/N sources and their cascading effects on microbial community dynamics in agroecosystems.

## Figures and Tables

**Table 1:** The TN and SOC concentrations and C/N ratios of different soil fractions in parental materials and straw incorporation treatments.

Treatments ^b^	Clay ^a^				Silt				Sand			
	Fraction Amount (%)	TN ^c^ (g kg^−1^)	SOC ^d^ (g kg^−1^)	C/N ^e^	Fraction Amount (%)	TN (g kg^−1^)	SOC (g kg^−1^)	C/N	Fraction Amount (%)	TN (g kg^−1^)	SOC (g kg^−1^)	C/N
CS_PM	34.9 (2.2)	0.19 (0.02) a	4.55 (0.08) a	24.1 (2.1) c	64.0 (1.2)	0.12 (0.03) a	2.48 (0.04) a	21.5 (2.2) b	1.1 (0.2)	0.07 (0.03) a	1.65 (0.03) a	26.9 (5.2) a
CS_L		0.81 (0.05) d	8.18 (0.11) d	10.1 (0.5) a		0.24 (0.06) b	3.32 (0.06) b	14.4 (3.4) a		0.09 (0.01) a	2.21 (0.04) b	24.7 (2.3) a
CS_M		0.61 (0.06) c	7.31 (0.10) c	12.0 (1.1) b		0.26 (0.05) b	3.98 (0.05) c	15.6 (2.8) a		0.09 (0.01) a	2.65 (0.03) c	29.6 (3.9) a
CS_H		0.52 (0.03) b	6.08 (0.15) b	11.7 (0.3) b		0.31 (0.07) b	4.46 (0.08) d	14.8 (3.2) a		0.12 (0.02) b	2.97 (0.05) d	25.1 (3.8) a
GS_PM	12.7 (1.5)	0.14 (0.02) a	3.52 (0.06) a	25.4 (3.2) c	45.1 (1.4)	0.07 (0.02) a	1.86 (0.03) a	28.0 (7.9) b	42.2 (3.1)	0.02 (0.01) a	0.41 (0.01) a	24.8 (13.5) a
GS_L		1.35 (0.11) d	13.07 (0.16) d	9.7 (0.7) a		0.25 (0.07) b	3.95 (0.07) b	16.6 (4.5) a		0.03 (0.01) a	0.65 (0.01) b	23.3 (7.8) a
GS_M		1.1 (0.03) c	11.66 (0.22) c	10.6 (0.2) b		0.31 (0.01) b	5.24 (0.10) c	16.9 (0.2) a		0.04 (0.01) b	1.16 (0.02) c	30.2 (7.2) a
GS_H		0.79 (0.08) b	8.79 (0.24) b	11.2 (0.8) b		0.39 (0.04) c	6.91 (0.13) d	17.8 (1.5) a		0.06 (0.02) b	1.53 (0.03) d	27.5 (9.1) a
PS_PM	10.6 (0.5)	0.56 (0.04) a	4.32 (0.08) a	7.7 (0.4) a	74.4 (1.7)	0.38 (0.03) b	2.02 (0.03) a	5.3 (0.3) a	15.0 (2.3)	0.25 (0.04) b	1.44 (0.02) a	5.8 (0.8) a
PS_L		0.82 (0.06) b	8.12 (0.22) b	9.9 (0.5) b		0.33 (0.05) ab	4.89 (0.10) d	15.0 (1.9) b		0.16 (0.02) a	3.49 (0.07) d	22.0 (2.3) b
PS_M		0.96 (0.07) c	9.74 (0.18) c	10.1 (0.6) b		0.29 (0.04) a	4.54 (0.08) c	15.8 (1.9) b		0.15 (0.02) a	3.24 (0.06) c	21.8 (2.5) b
PS_H		1.06 (0.09) d	10.47 (0.15) d	9.9 (0.7) b		0.26 (0.08) a	3.79 (0.07) b	15.5 (4.7) b		0.13 (0.03) a	2.71 (0.05) b	21.5 (4.7) b

^a^ Clay, <2 μm size fraction; silt, 2–63 μm size fraction; sand, 63–2000 μm size fraction. ^b^ CS, Quaternary red clay soil; GS, granite soil; PS, purple sandy shale; PM, parental material; L, low-C/N-ratio straw incorporation; H, high-C/N-ratio straw incorporation; M, mixed-straw incorporation with low- and high-C/N-ratio straws. ^c^ TN, total N in clay, silt, and sand fractions, respectively. ^d^ SOC, soil organic C in clay, silt, and sand fractions, respectively. ^e^ C/N, the ratio of soil total C and total N. Numbers inside the parentheses indicate the standard deviations. Different letters in column indicate significant differences (*p* < 0.05) between parental material and treatments within the same type of soil according to Duncan’s multiple-comparison test.

**Table 2:** Spearman’s rank correlation between the abundance of *nifH* gene and diazotrophic bacteria and soil properties.

Soil Property	Clay ^a^		Silt		Sand	
	TN ^b^	SOC ^c^	TN	SOC	TN	SOC
*nifH* gene ^d^	0.762 **	0.819 **	0.424 *	0.557 *	−0.184	0.216
diazotrophic bacteria ^e^	0.885 **	0.903 **	0.341	0.475 *	−0.232	0.169

^a^ Clay, <2 μm size fraction; silt, 2–63 μm size fraction; sand, 63–2000 μm size fraction. ^b^ TN, total N in clay, silt, and sand fractions, respectively. ^c^ SOC, soil organic C in clay, silt, and sand fractions, respectively. ^d^ The *nifH* gene abundance. ^e^ The relative abundance of diazotrophic bacteria. Asterisks reveal significance at *p* < 0.05 (*) and *p* < 0.01 (**).

## Data Availability

The original contributions presented in this study are included in the article/Supplementary Materials. Further inquiries can be directed to the corresponding author.

## Supplementary Materials

The following supporting information can be downloaded at: https://www.mdpi.com/article/10.3390/microorganisms13051104/s1, Table S1: The genes in the organic carbon metabolism process.
