# Supplementary material for: Soil Native C/N Ratio Affects Diazotrophic Bacterial Composition and N Fixation by Regulating SOC Distribution in Soil Particles After Residue Incorporation
Source: Microorganisms. 2025 May 11;13(5):1104. doi: 10.3390/microorganisms13051104 (PMC12114343; doi:10.3390/microorganisms13051104)
Supplement: Supplementary file 1 [file microorganisms-13-01104-s001.zip › microorganisms-3600328-supplementary.pdf]

Table S1. The genes of organic carbon metabolism process.

Readily Organic Carbon Metabolism Process					Recalcitrant Organic Carbon Metabolism Process	
K00024	K00170	K00247	K01057	K01690	K00023	K01846
K00030	K00171	K00382	K01616	K01783	K00140	K01848
K00031	K00172	K00615	K01623	K01803	K00232	K01849
K00033	K00174	K00616	K01624	K01807	K00248	K02437
K00036	K00175	K00627	K01625	K01808	K00261	K03417
K00116	K00176	K00658	K01647	K01810	K00281	K03781
K00131	K00177	K00845	K01659	K01834	K00600	K04835
K00134	K00239	K00850	K01676	K01902	K00605	K05605
K00150	K00240	K00873	K01677	K01903	K00626	K08691
K00161	K00241	K00874	K01678	K03737	K00830	K09788
K00162	K00242	K00886	K01679		K01601	K11263
K00163	K00244	K00918	K01681		K01602	K11517
K00164	K00245	K00927	K01682		K01638	K14446
K00169	K00246	K00948	K01689		K01720	K14447
K15916	K13831	K08093	K03738		K15919	K14448
K16306	K15633	K08094	K05308		K17865	K14451
K16370	K15634	K11645	K06859		K18472	K15918
K18118	K15635	K13810	K07404		K19268	
